# Revisiting the dangers of the coronavirus in the ophthalmology practice

**DOI:** 10.1038/s41433-020-0790-7

**Published:** 2020-02-06

**Authors:** Ivan Seah, Xinyi Su, Gopal Lingam

**Affiliations:** 1 Department of Ophthalmology, National University Hospital, Singapore, Singapore; 2 Institute of Molecular and Cell Biology (IMCB), Agency for Science, Technology and Research (A*STAR), Singapore, Singapore; 3 Department of Ophthalmology, Yong Loo Lin School of Medicine, National University Singapore, Singapore, Singapore

**Keywords:** Microbiology, Viral infection

## Introduction

Every year, during the Lunar New Year, the largest human migration in the world occurs in China. Almost 3 billion passenger-journeys are made as travellers reunite with distant families [1]. This year, things are different. The recent emergence of a novel coronavirus (2019-nCoV), which caused an outbreak of viral pneumonia in Wuhan, China, has led to three Chinese cities; Wuhan, Huanggang, Ezhou, placed under lockdown to curb transmission. The 2019-nCoV has sparked global concern regarding the likelihood of the epidemic turning out like the 2003 Severe Acute Respiratory Syndrome Coronavirus (SARS-CoV), where more than 8000 people were infected with 774 mortalities [2]. Healthcare workers represented 20% of the infected [2]. The 2019-nCoV serves as a reminder of the potential dangers posed by coronaviruses to both patients and doctors alike.

## Coronaviruses: what are they?

**Fig. 1:**
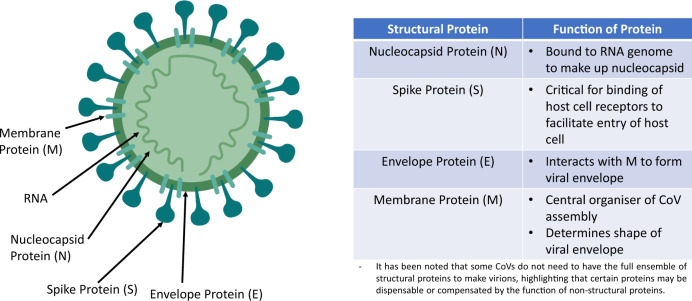
Main structure of coronaviruses.

Coronaviruses (CoV) belong to the subfamily *Coronavirinae*, in the family *Coronaviridae* of the order *Nidovirales*. There are four genera: *Alphacoronavirus, Betacoronavirus, Gammacoronavirus* and *Deltacoronavirus* [3]. It is a single positive-sense RNA virus. Mutation rates of RNA viruses are greater than DNA viruses, suggesting a more efficient adaptation process for survival. The genome codes for at least four main structural proteins: spike (S), membrane (M), envelope (E), nucleocapsid (N) proteins and other accessory proteins which aid the replicative processes and facilitate entry into cells [4]. Figure 1 summarises the coronavirus’s structure and the function of the structural proteins. CoVs mainly affect birds and mammals. Prior to 2019, there were only six CoVs that can infect human and cause respiratory diseases: HCoV-229E, HCoV-OC43, HCoV-NL63, HKU1, SARS-CoV, MERS-CoV. The last 2 are capable of causing severe respiratory syndrome in humans.

## The Wuhan Novel Coronavirus (2019-nCoV)

On the 31st of December 2019, the World Health Organisation (WHO) was alerted by Chinese authorities of a series of pneumonia-like cases in the city of Wuhan, a city the size of London with about 11 million people. It was quickly identified that the first human infections likely originated from Huanan Seafood Market in Wuhan [5]. Two weeks later, a group of Chinese scientists, along with WHO announced that a new coronavirus (2019-nCoV), identified through genomic sequencing, was the culprit of the pneumonia [6]. Symptoms of infection included fever, malaise, dry cough, shortness of breath and respiratory distress.

While such an effort is a crucial response to tackling the crisis, understanding of the virus’s transmission patterns still remain murky. Initially thought to be a virus with mainly animal-human transmission, this was proven to be untrue when the number of cases surged over the weekend of 18th and 19th January and reports of healthcare workers being infected surfaced [7]. As of the 23rd of January 2020, 622 have been infected globally with 17 mortalities all located within China.

**Fig. 2:**
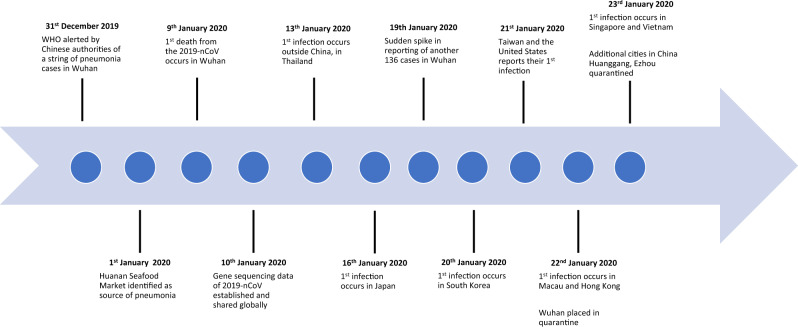
Key events in the 2019-nCoV outbreak.

A timeline of key events up till the 23rd of January is represented in Fig. 2.

## A possible threat in the ophthalmology clinic

While the 2019-nCoV transmission route is still unknown, countries have been preparing measures based on past experiences with coronaviruses namely SARS-CoV and MERS-CoV. These viruses transmit primarily through droplets and other bodily secretions. In the ophthalmology practice, healthcare workers may be particularly susceptible to these infections.

Firstly, ophthalmologists are extremely reliant on physical examination during patient consultation. Of particular concern is the proximity between the patient and ophthalmologist during the slit lamp microscope examination. It has been shown that droplets from a cough or sneeze can be propelled for up to 6 m [8], a range that definitely encompasses the distance between the patient and ophthalmologist.

Secondly, during the SARS-CoV epidemic, clinical reports have suggested tears as a medium of infection. In a case series by Loon et al., it was shown that viral RNA of the SARS-CoV can be detected by reverse-transcription polymerase chain reaction (RT-PCR) from the tears of infected individuals [9]. While anecdotal in nature, such accounts highlight the possible infectivity of tears, a fluid which ophthalmologists and instruments come in contact on a daily basis. If true, this represents a crucial need for further development of disinfection and personal protective equipment (PPE) protocols for the ophthalmology clinic.

## Bringing back strategies to prevent transmission

In view of the potential threat in the ophthalmology practice, it may be prudent to revisit the strategies that successfully curbed the transmission of SARS in 2003 [10]. With particular relevance to the ophthalmic practice, it may be beneficial to triage patients according to produced surveillance case definitions [11]. In 2003, the WHO launched a case classification scheme which triaged patients into general, suspect and probable categories. Ophthalmology practices in Hongkong, a country badly hit by SARS, recommended the full PPE for all cases regardless of SARS status. For suspect and probable cases, appointments were recommended to be deferred unless in the event of an ophthalmic emergency. These patients were seen in an isolation ward. An emphasis on hand hygiene measures and stocking up of PPE such as N95 masks, gloves, gowns and googles should also be considered while the mode of transmission is being identified. Decontamination and sterilisation protocols of clinical rooms and equipment should also be improved on as coronaviruses have been found to survive in environments outside the body for a long time [12]. For instance, it is still not established if higher concentrations of dilute bleach (1:10), a chemical used to sterilise the Goldmann applanation tonometer, can be utilised to eliminate coronaviruses [13]. Other shared equipment like the B-scan probe and contact lenses for photocoagulation will also need strict sterilisation protocols. Finally, the reduction of non-urgent ophthalmic operations should also be considered as the risk of viral transmission may outweigh the surgical benefits. For emergency operations, full PPE can be considered to reduce the probability of healthcare transmission.

As the WHO Director-General, Tedros Adhanom Ghebreyesus highlighted, “Make no mistake: this is an emergency in China. But it has not yet become a global health emergency. It may yet become one.” It is currently very difficult to predict the eventual impact of the 2019-nCoV. However, it will be prudent to utilise the lessons gained from SARS-CoV and prepare for the worst. Until a vaccine is created, public health strategies are likely the best weapons against the enemy.
